# Effects of Excimer Laser Treatment of Zirconia Disks on the Adhesion of L929 Fibroblasts

**DOI:** 10.3390/ma16010115

**Published:** 2022-12-22

**Authors:** Yoshihiko Akashi, Yoshiaki Shimoo, Hayato Hashiguchi, Kei Nakajima, Katsutoshi Kokubun, Kenichi Matsuzaka

**Affiliations:** 1 Department of Pathology, Tokyo Dental College, 2-9-18 Kandamisaki-cho, Chiyoda-ku, Tokyo 101-0061, Japan; 2 MALO DENTAL and MEDICAL TOKYO, FUKUHARA GINZA 8F, 7-8-10 Ginza, Chuo-ku, Tokyo 104-0061, Japan

**Keywords:** excimer laser, zirconia, adhesion, fibroblast, photofunctionalization, implant surface

## Abstract

The adhesion of zirconia and soft tissue is very important for the success of zirconia implants. The aim of this study was to characterize the effects of excimer laser treatment of zirconia on the adhesion of L929 fibroblasts. In this study, polished zirconia disks treated with an excimer laser were the experimental group and untreated zirconia disks were the control group. Surface roughness and contact angles of zirconia disks were measured. mRNA expression levels of integrin β1 and collagen type I α1 in L929 fibroblasts cultured on zirconia disks were measured using qRT-PCR. Cell morphology was evaluated using 3D laser microscopy and the expression of vinculin was characterized using confocal microscopy. There was no significant difference in the surface roughness of zirconia disks, but contact angles were significantly lower. mRNA expression of integrin β1 was significantly higher at 3, 6 and 24 h and of collagen type I α1 was significantly higher at 6 and 24 h. L929 fibroblasts tended to form elongated microspikes and vinculin colocalization in those microspikes. Furthermore, vinculin was strongly expressed in filopodia of L929 fibroblasts at 24 h. These results suggest that excimer laser treatment improves adhesion between zirconia disks and L929 fibroblasts.

## 1. Introduction

The standard material used for dental implant systems is titanium, but some studies have reported that when titanium releases trace amounts of metal ions in the body, it can cause adverse effects such as metal allergies [1,2]. Moreover, there is an aesthetic problem since titanium implants shine through thin gingiva due to their gray coloring. To solve those problems, ceramics such as zirconia are now being introduced as alternative materials for dental implant systems. Zirconia, especially tetragonal zirconia polycrystal (TZP), has the advantage of improved mechanical performance, biocompatibility, and aesthetic appearance [3,4,5]. Therefore, zirconia dental implants not only avoid metal allergies but also reduce the shining of implants through thin gingival mucosa, providing excellent aesthetics, especially in dental implant abutments.

The main cause of dental implant failure is peri-implantitis infections because of the penetration of bacterial plaques into the peri-implant sulcus. Therefore, for the long-term success of dental implants, it is important not only to obtain osseointegration between the implant and the bone, but also to obtain an effective biological seal against microorganisms between the abutment and the gingival soft tissue [6,7]. To achieve soft-tissue sealing, fibroblasts, which are regarded to as the main cell type in peri-implant soft tissue, need to adhere to the abutment. To obtain better adhesion between the material surface and the fibroblasts, several in vitro studies have investigated the relationship between material surface hydrophilicity and cell adhesion [8,9]. High surface wettability, which implies high surface energy, is generally reported to promote greater cell adhesion than low surface wettability. Superhydrophilicity has been reported to enhance cellular functions. Superhydrophilicity can be obtained by ultraviolet (UV) irradiation and low-temperature plasma treatments such as glow discharge and is known as photofunctionalization [10,11]. It has been reported that photofunctionalization by UV treatment or plasma treatment improves the adhesion between titanium and zirconia, which are materials used in dental implants, and various cells such as osteoblasts [12,13], epithelial cells [14,15,16] and fibroblasts [17,18].

Currently, in clinical practice, photofunctionalization by UV treatment using low-pressure mercury lamps is used to improve the adhesion between dental implant bodies and cells of the surrounding tissue. Furthermore, excimer laser treatment is attracting attention as a new approach to photofunctionalization. An excimer laser lamp externally excites xenon, which is one of the noble gases and irradiates the laser with a light energy of 7.21 eV, which corresponds to a wavelength of 172 nm, which is shorter than conventional UV lasers. Conventional UV lamps emit wavelengths by evaporating mercury in the lamp tube due to the temperature rise associated with the discharge of the filament electrode, so the illuminance of the lamp increases with temperature and reaches the maximum illuminance. On the other hand, an excimer UV lamp does not increase in illuminance with temperature, so it reaches the maximum illuminance about 2 s after being turned on.

It has been reported that the physical properties of zirconia are changed by treatment with an excimer laser [19,20,21]. However, there are no reports yet of cell dynamics on zirconia surfaces after excimer laser treatment. The aim of this study was to investigate the effects of excimer UV treatment of zirconia on the adhesion of L929 fibroblasts.

## 2. Materials and Methods

### 2.1. Specimen Preparation and Surface Treatment

Zirconia disks were used in this study. Disks of 13 mm diameter and 0.5 mm thickness were prepared using a cutting machine (FINE CUT, HS-100 G-type, Heiwa Technica, Tokyo, Japan). They were ground progressively finer down to 1200 grit and then were finely polished with 9 µm and 3 µm diamond paste and 0.02 µm colloidal silica using a polishing machine (Ecomet 250/Automet 250, Buehler, IL, USA). Subsequently, the disks were ultrasonically cleaned with acetone and distilled water and then were sterilized in an autoclave for 10 min at 121 °C after cleaning.

In the experimental group, the disks were irradiated with an excimer laser. In the control group, the disks were not irradiated with an excimer laser. Excimer UV treatment was performed using a Super Osseo Integration Excimer UV system (E172-110, Excimer, Inc., Kanagawa, Japan) at room temperature for 10 min. This equipment creates Excimer UV radiation with a total power of 20 mW/cm^2^ and an excitation wavelength of 172 nm.

### 2.2. Surface Roughness and Surface Wettability

The arithmetic surface roughness (Sa) of the zirconia disks was measured using a 3D laser measuring microscope (LEXT OLS4100, Olympus, Tokyo, Japan) with a length of 4 mm and a cut-off value of 0.8 mm.

The surface wettability of the zirconia disks was assessed by measuring the contact angle using a contact angle meter (Phoenix α, Meiwa-forces, Tokyo, Japan) at 3 s after application of each droplet of 4 μL distilled water.

We were measured five zirconia disks each for unpolished, polished, polished with excimer laser treatment. 

### 2.3. Cell Culture

Cell culture experiments were performed using L929 fibroblasts (RIKEN BRC CELL BANK, Tsukuba, Japan). Cells were cultured at 37 °C in a CO_2_ incubator (5%) in DMEM (High glucose) (NACALAI TESQUE, INC., Kyoto, Japan) with 10% fetal bovine serum (FBS) (Biowest, Nuaillé, France). The medium was renewed every three days. When cells reached 80–90% confluency, they were detached using 0.05% trypsin EDTA (Gibco BRL, Grand Island, NY, USA) and were seeded onto zirconia disks at a density of 4 × 10^4^/cm^2^. In this experiment, zirconia disks with excimer treatment after polishing were used in the experimental group and zirconia disks without excimer treatment after polishing was used in the control group.

### 2.4. Quantitative RT-PCR

mRNA expression levels of 2 cell attachment proteins, integrin β1 and collagen type I α1, on each disk were measured by quantitative RT-PCR. L929 fibroblasts at 3, 6 and 24 h in each group were collected using a cell scraper. Total RNAs were extracted from L929 fibroblasts using an RNeasy Mini Kit (QIAGEN, Hilden, Germany) and were reverse-transcribed into complementary DNAs (cDNAs) using ReverTra Ace qPCR RT Master Mix with gDNA Remover (TOYOBO, Osaka, Japan).

Quantitative RT-PCR was performed using a 7500 Fast Real-Time PCR System (Applied Biosystems, Bedford, MA, USA) in TaqMan Gene Expression Assays (Applied Biosystems) to determine the expression of integrin β1 mRNA (Itgb1, Mm01253230_m1), collagen type I α1 mRNA (Col1a1, Mm00801666_g1) and glyceraldehyde-3-phosphate dehydrogenase mRNA (Gapdh, Mm99999915_g1) used as an endogenous control. Reaction conditions consisted of a primary denaturation at 95 °C for 20 s and then cycling for 40 cycles of 95 °C for 3 s and 62 °C for 30 s. Each mRNA expression level was corrected based on the Gapdh mRNA expression level, and target gene expression levels were subjected to relative quantitative analysis.

### 2.5. Cell Morphometry

At 3, 6 and 24 h after seeding, the disks were washed three times with PBS to remove the culture medium and then were fixed in 10% paraformaldehyde for 30 min at room temperature. The cells were then washed three times in PBS. The samples were observed and imaged using a 3D laser measuring microscope (LEXT OLS4100, Olympus).

### 2.6. Immunofluorescence Observations

To observe the subcellular distribution of vinculin, cells were washed in PBS after 3, 6 and 24 h of cultivation and then were fixed in 10% paraformaldehyde for 30 min at room temperature. The cells were then washed three times in PBS, after which nonspecific binding was blocked with 1% fetal bovine serum (FBS) for 30 min at room temperature.

To observe actin filaments, the cells were incubated for 30 min at room temperature with FITC-conjugated phalloidin (1:100 dilution, Invitrogen, Waltham, MA, USA) or were incubated overnight at 4 °C with a primary rabbit anti-vinculin antibody (1:100 dilution, Invitrogen, Waltham, MA, USA). After three additional washes in PBS, the samples were incubated with a secondary antibody, Alexa fluor 488 goat anti-rabbit immunoglobulin G (IgG) (1:100 dilution, Invitrogen, Waltham, MA, USA), which was detected as green fluorescence for 30 min at room temperature. After five washes in PBS, a micro cover glass was placed over each sample overnight for 4 °C with ProLong with DAPI (Invitrogen, Waltham, MA, USA).

These samples were observed using a confocal laser scanning microscope (LSM 880 Airy NLO) with software (Zen, Carl Zeiss, Oberkochen, Germany).

### 2.7. Statistical Analysis

Statistical analysis was performed using Mann-Whitney analysis and one-way analysis of variance (ANOVA) at each culture period.

## 3. Results

### 3.1. Surface Roughness and Surface Wettability

**Figure 1:**
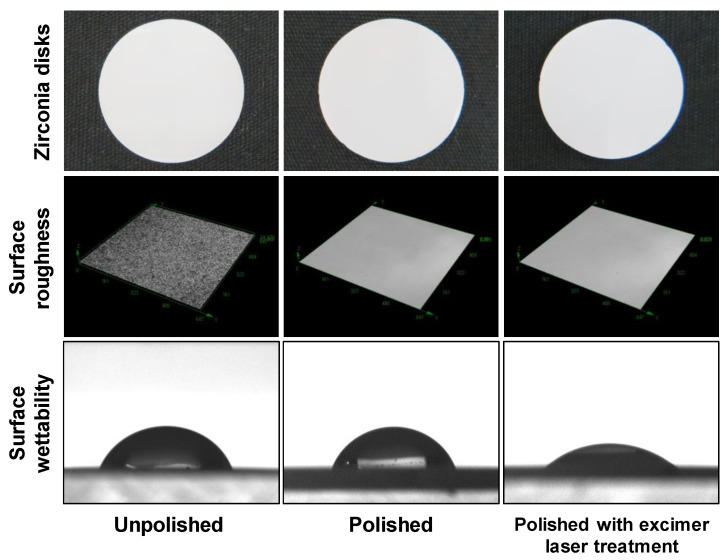
Surface roughness and surface wettability of zirconia disks. Macroscopically, there was no difference in the surface properties of the zirconia discs. In a 3D measuring laser microscope, the surface texture was smoother in the polished disks compared to the unpolished disks, but there was no difference in surface texture between the polished disks with or without the excimer laser treatment. There was no difference in surface wettability between the unpolished and polished disks, but the polished disks with the excimer laser treatment had an improved surface wettability compared to the polished disks without the excimer laser treatment.

Macroscopically, there was no difference in the surface properties of the zirconia discs. In a 3D measuring laser microscope, the surface texture was smoother in the polished disks compared to the unpolished disks, but there was no difference in surface texture between the polished disks with or without the excimer laserexcimer laser treatment. There was no difference in surface wettability between the unpolished and polished disks, but the polished disks with the excimer laser treatment had an improved surface wettability compared to the polished disks without the excimer laser treatment (Figure 1).

The Sa value of the zirconia disks was 0.0078 ± 0.002 µm in the polished disks irradiated with an excimer laser, 0.0080 ± 0.0003 µm in the polished disks without the excimer laser treatment, and 0.1610 ± 0.0345 µm in the unpolished disks (Table 1).

**Figure 2:**
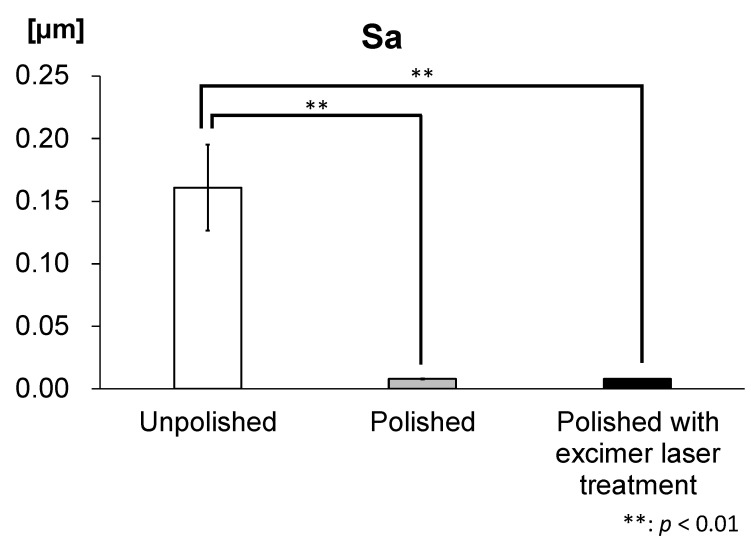
Arithmetic surface roughness (Sa) of zirconia disks. The Sa value was significantly lower in polished disks and in polished disks irradiated with an excimer laser compared to unpolished disks. (*n* = 5).

The Sa value was significantly lower in the polished disks and in the polished disks irradiated with an excimer laser compared to the unpolished disks (Figure 2).

The contact angle of zirconia disks was 33.1° ± 7.8 in polished disks irradiated with an excimer laser, 55.3° ± 2.5 in polished disks and 50.5° ± 4.8 in unpolished disks (Table 2). 

**Figure 3:**
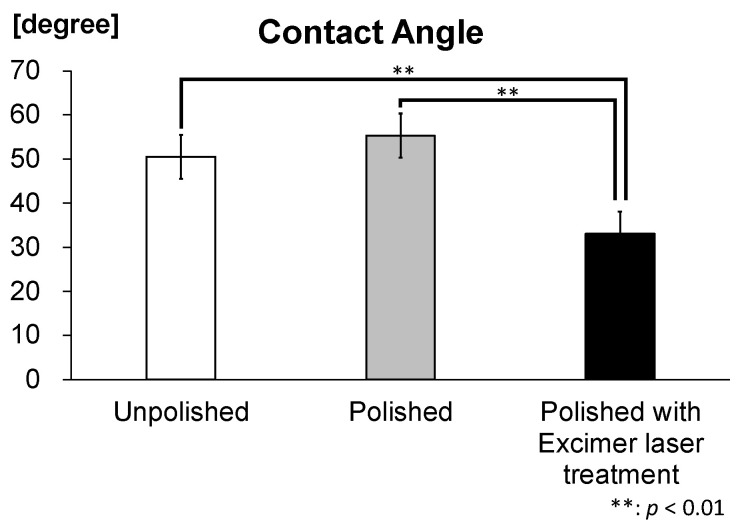
Contact angle of zirconia disks. The contact angle of polished disks irradiated with the excimer laser was significantly lower than the unpolished disks and polished disks. (*n* = 5).

The contact angle of polished zirconia disks irradiated with Excimer laser was significantly lower than unpolished disks and polished disks, resulting the superhydrophilicity (Figure 3).

### 3.2. Quantitative RT-PCR

**Figure 4:**
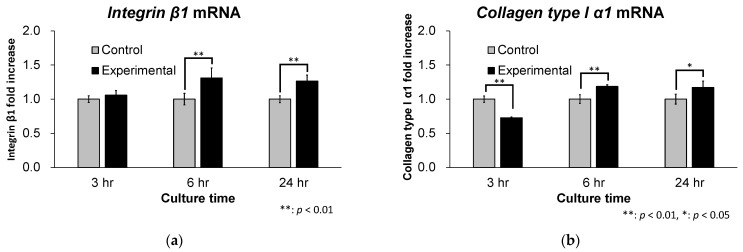
mRNA expression levels of Integrin β1 and Collagen type I α1. (**a**) The mRNA expression level of integrin β1 was significantly higher in the experimental group compared to the control group at 6 and 24 h. (*n* = 5) (**b**) The mRNA expression level of collagen type I α1 was significantly higher in the experimental group compared to the control group at 6 and 24 h. (*n* = 5).

The mRNA expression level of integrin β1 at 3, 6 and 24 h is shown in Figure 4a. There was no significant difference between the 2 groups at 3 h but it was significantly higher in the experimental group compared to the control group at 6 and 24 h. The mRNA expression level of integrin β1 in the experimental group at 6 and 24 h was approximately 1.3 times greater than was that in control groups at 3 h. The mRNA expression level of collagen type I α1 at 3, 6 and 24 h is shown in Figure 4b. Those levels were significantly lower in the experimental group compared to the control group at 3 h, but they were significantly higher in the experimental group compared to the control group at 6 and 24 h. The mRNA expression level of collagen type I α1 in the experimental group at 6 and 24 h was approximately 1.2 times greater than that in control groups at 3 h.

### 3.3. Cell Morphometry and Immunofluorescence Observations

**Figure 5:**
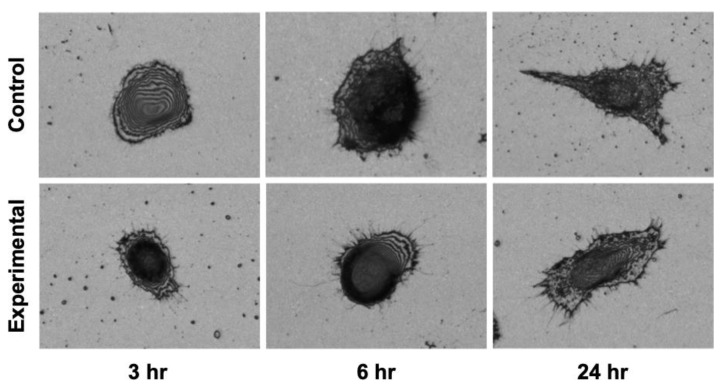
Cell morphology assessed using a 3D measuring laser microscope. The cell morphology showed a trend toward slightly thicker microspike formation and spike elongation in the experimental group compared to the control group.

3D measurement laser microscopy observations revealed the cell morphology and elongated microspike formation in L929 fibroblasts (Figure 5). There was no clear difference in cell morphology at 3, 6 and 24 h. However, at 3 h, elongated microspike formation was observed predominantly in the experimental group compared to the control group. After that, the microspikes tended to elongate with time. Microspike formation was also observed in the control group at 6 and 24 h as in the experimental group.

**Figure 6:**
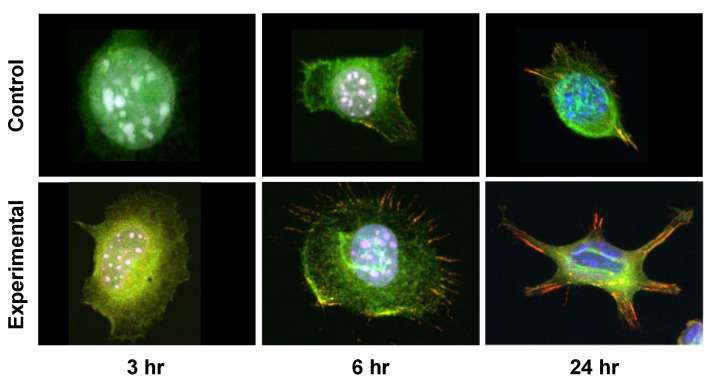
Immunocytochemistry of vinculin. Immunocytochemistry reveals vinculin expression within the elongated microspikes of fibroblasts. Furthermore, the elongation of vinculin-expressing thick spikes was observed at 24 h. (Vinculin: red, DAPI: blue, Phalloidin: green).

Immunocytochemistry revealed vinculin expression within elongated microspikes of L929 fibroblasts (Figure 6). At 3 h, no vinculin expression was observed in either the control or the experimental groups. However, broader cytoplasmic staining was observed in the experimental group compared to the control group. At 6 h, vinculin expression was observed consistent with microspikes radiating from the cytoplasmic margin of L929 fibroblasts in the experimental group. In the control group, little vinculin expression was observed at 6 h. At 24 h, L929 fibroblasts formed filopodia-like structures in the experimental group, and vinculin was strongly expressed in the filopodia. In the control group, vinculin expression was observed at the cytoplasmic margin at 24 h.

## 4. Discussion

In this study, we investigated the response of L929 fibroblasts cultured on zirconia surfaces that were treated with an excimer laser for photofunctionalization. The results showed that the attachment capability of L929 fibroblasts was enhanced on zirconia disks, treated with the excimer laser compared with the untreated disks. Specifically, the mRNA expression levels of integrin β1 and collagen type I α1 in the experimental group were significantly higher than in the control group. Furthermore, in the experimental group, elongated microspikes and filopodia-like structures were observed in L929 fibroblasts cultured on zirconia discs, and vinculin was expressed inside those cells.

Several reports have revealed that cell attachment is promoted on superhydrophilic surfaces compared to hydrophobic surfaces [9,22,23]. Photofunctionalization is a method to improve the wettability of zirconium and titanium used as abutments in dental implant systems and enhances their adhesion to the surrounding cells. In previous reports, zirconia and titanium were photofunctionalized using UV and plasma treatments and had improved wettability. However, there is also a report that the photofunctionalization of zirconia using UV treatment did not show a significant difference in wettability [15]. Photocatalytic activity is believed to be closely involved in differences in the formation of basic hydroxyl groups and the polar component. In the case of titanium, UV light energy above 3.2 eV, which corresponds to wavelengths below 387 nm, is required to trigger the photocatalytic activity of TiO_2_ to excite electrons from the valence band to the conduction band [24]. ZrO_2_ also exhibits a photocatalytic activity similar to TiO_2_ [25,26]. ZrO_2_ has a bandgap of 5.82 eV, which corresponds to a wavelength of approximately 213 nm. Therefore, more energy is required to induce photocatalytic activity in ZrO_2_ than in TiO_2_. Clinically, the photofunctionalization of conventional implants may be stimulated with a UV device with a light energy of 4.9 eV, which corresponds to a wavelength of 254 nm. This UV treatment provides sufficient light energy for the photofunctionalization of titanium but may not provide enough light energy for photofunctionalization of zirconia. The excimer laser device used in this study is a Xe excimer UV lamp with a light energy of 7.21 eV, which corresponds to a wavelength of 172 nm, which is expected to photofunctionalize zirconia. In this study, there was no difference in the contact angle of zirconia discs before and after polishing. However, the contact angle of zirconia disks was smaller in the excimer laser irradiation group compared to the non-irradiation group, and the wettability was improved. This suggests that the photofunctionalization of zirconia provided hydrophilicity. The O_2_-plasma device is capable of producing very high optical energies of 5.0 eV to 13.1 eV, which corresponds to wavelengths of 95 nm to 250 nm and poses no problem for the photofunctionalization of zirconia. However, the clinical application of this device is very difficult because it is very large. On the other hand, the excimer laser device is small and is very effective for the clinical application of photofunctionalization. In addition, the short wavelength of the laser means that the energy of the light is large and can be irradiated efficiently. In other words, it leads to shortening the irradiation time, and a typical UV treatment takes about 12–15 min [27,28]. Additionally, it takes several min for the irradiator to reach maximum illuminance after it is turned on. The excimer laser device reaches its maximum illuminance in about 2 s after turning on the power. For this reason, in clinical practice, photofunctionalization by excimer laser irradiation takes less time than conventional light sources. In addition, since conventional UV lamps use mercury, they may be affected by the International Minamata Convention, which regulates the manufacture and sale of products containing mercury. Therefore, we suggest that the photofunctionalization of zirconia by excimer laser treatment has a great clinical benefit.

Adhesion is modulated by the engagement, clustering and turnover of adhesion receptors. Integrins are one type of adhesion receptor and are prominently concentrated in matrix adhesions, such as focal complexes and focal adhesions [29]. Focal complexes are small transient adhesions at the cell periphery, which grow in size to become focal adhesions, which are larger, more stable structures. The key to the function of adhesion receptors is the recruitment of proteins that link the adhesion receptor to the actin cytoskeleton. While many proteins are involved in that process, vinculin, a cytoplasmic actin-binding protein enriched both at cell-cell and at cell-matrix adhesions, is one of the best characterized. Vinculin was identified nearly 40 years ago in the laboratories of Benjamin Geiger and Keith Burridge as a component of focal adhesion and adherence junctions [30,31]. Cell-matrix adhesions of focal adhesions are rich in adhesion receptors known as integrins. More than 50 different proteins are known to be recruited to the integrin cytoplasmic tail [32]. In the leading edge of cells, vinculin is localized to newly formed adhesions [33]. The nascent adhesions are rich in vinculin bound to the Arp2/3 complex, a potent nucleator of action polymerization [34]. This interaction localizes actin polymerization to the newly formed adhesions and enables connections between integrins and the actin polymerization machinery. These newly formed adhesions trigger further protrusions of the cell membrane [34,35,36]. Matsuzaka et al. revealed that vinculin is an important protein in the adhesion of materials involved in focal adhesions [37]. In this study, 3D laser microscopy images showed that microspikes formed at the periphery of L929 fibroblasts over time and elongated microspikes, especially in the experimental group. Immunocytochemical staining with confocal microscopy showed vinculin expression radially, consistent with elongated microspikes in the experimental group. Furthermore, vinculin was strongly expressed in filopodia observed at 24 h in the experimental group. This result suggests that cell adhesion is improved by the formation of focal adhesions at the edges of L929 fibroblasts in the experimental group. Vinculin plays a role in maintaining focal adhesions. This study reveals that fibroblast adhesions achieve an effective biological seal to the abutment of dental implants, which we call “fibroadhesions”. The recruitment of vinculin to talin stabilizes focal adhesions and promotes integrin clustering and enlargement [38,39]. More recent work has shown that vinculin directly regulates integrin activation through talin [39,40,41]. Indeed, the qRT-PCR analysis in this study showed that the mRNA expression level of integrin β1 increased over time in the experimental group. In addition, the mRNA expression level of collagen type I α1, which encodes collagen type I and plays a role in improving the adhesion between cells and materials, was lower at 3 h, but was higher at 6 and 24 h in the experimental group. This result suggests that stronger focal adhesions are maintained between zirconia and L929 fibroblasts in the experimental group.

## 5. Conclusions

Excimer laser irradiation improved the wettability of zirconia discs, and it was expected that the adhesion between zirconia and cells would be improved. In this study, the integrin β1 and collagen type I α1 mRNA expression level of L929 fibroblast in the experimental group were significantly higher than those in the control group at 6 and 24 h. Furthermore, morphologically, L929 fibroblast formed filopodia-like structures, and vinculin expression was observed in agreement with the pseudopodia at 24 h. These results suggest that the excimer laser treatment of zirconia disks improves the adhesion of L929 fibroblasts. Therefore, it provided a barrier against microbial infection between the zirconia implant and the soft tissue cells and is expected to prevent implant dislodgment due to bacterial infection.

## Figures and Tables

**Table 1:** Surface roughness values of zirconia disks. Data represent means (± SD).

Zirconia Disk	Surface Roughness (Sa, mm)
Unpolished	0.1610 ± 0.0345
Polished	0.0080 ± 0.0003
Polished with excimer laser treatment	0.0078 ± 0.002

**Table 2:** Contact angle values of zirconia disks. Data represent means (± SD).

Zirconia Disk	Contact Angle
Unpolished	50.5° ± 4.8
Polished	55.3° ± 2.5
Polished with excimer laser treatment	33.1° ± 7.8

## Data Availability

Not applicable.
